# Structure of the Malaria Antigen AMA1 in Complex with a Growth-Inhibitory Antibody

**DOI:** 10.1371/journal.ppat.0030138

**Published:** 2007-09-28

**Authors:** Andrew M Coley, Aditi Gupta, Vince J Murphy, Tao Bai, Hanna Kim, Robin F Anders, Michael Foley, Adrian H Batchelor

**Affiliations:** 1 Cooperative Research Center for Diagnostics, Department of Biochemistry, La Trobe University, Victoria, Australia; 2 Department of Biochemistry, La Trobe University, Victoria, Australia; 3 University of Maryland School of Pharmacy, Baltimore, Maryland, United States of America; Institut Pasteur, France

## Abstract

Identifying functionally critical regions of the malaria antigen AMA1 (apical membrane antigen 1) is necessary to understand the significance of the polymorphisms within this antigen for vaccine development. The crystal structure of AMA1 in complex with the Fab fragment of inhibitory monoclonal antibody 1F9 reveals that 1F9 binds to the AMA1 solvent-exposed hydrophobic trough, confirming its importance. 1F9 uses the heavy and light chain complementarity-determining regions (CDRs) to wrap around the polymorphic loops adjacent to the trough, but uses a ridge of framework residues to bind to the hydrophobic trough. The resulting 1F9-AMA1–combined buried surface of 2,470 Å^2^ is considerably larger than previously reported Fab–antigen interfaces. Mutations of polymorphic AMA1 residues within the 1F9 epitope disrupt 1F9 binding and dramatically reduce the binding of affinity-purified human antibodies. Moreover, 1F9 binding to AMA1 is competed by naturally acquired human antibodies, confirming that the 1F9 epitope is a frequent target of immunological attack.

## Author Summary

Malaria caused by Plasmodium falciparum causes more than 1 million deaths annually, and the development of a vaccine against this parasite is a major public health priority. Development of a vaccine is considered feasible because infection with malaria parasites induces protective immune responses, which include antibodies to a range of proteins on the parasite surface. Antigenic diversity allows the parasite to evade protective responses, and this may make it difficult to develop a vaccine that is effective against most infections. To facilitate the design of an effective vaccine, a more detailed understanding of how antibodies interact with their target parasite antigens is required. Here, we provide a detailed structural picture of the interaction between a growth-inhibitory monoclonal antibody and the leading vaccine candidate, AMA1. The results provide important insights into why some antibodies are inhibitory and why antigenic diversity in AMA1 enables the parasite to evade protective antibody responses.

## Introduction

Malaria is a global health problem that results in up to 3 million deaths annually [1,2]. Most at risk are young children living in malaria-endemic regions. Older children develop immunity to the parasite such that there is a reduction in parasite densities and the associated morbidity and mortality [3]. Studies demonstrating protection from passive immunization suggest that a significant component of acquired protective immunity is antibody-mediated [4–6]. Identifying the antigens recognized by protective immune responses induced by malaria has been difficult, but many proteins associated with the merozoite surface or apical organelles are targets of antibodies that block merozoite invasion.

One such antigen that shows promise as a vaccine candidate is apical membrane antigen 1 (AMA1). AMA1 is a type I integral membrane protein with a 55–amino acid cytoplasmic segment and a 550–amino acid extracellular region that can be divided into three domains on the basis of intradomain disulphide bonds [7]. Recombinant AMA1 ectodomain is highly effective at inducing protection in animal models of human malaria [8,9]. Protection by passive transfer in mice [10] and the absence of protection in B cell–deficient mice [11] suggest an important role for the humoral immune response. Refolded recombinant AMA1 induces protection, whereas no protection is induced by reduced and alkylated AMA1 [9,10]. Thus, protection induced by AMA1 is mediated by antibodies that recognize conformational epitopes on the surface of the protein. Rabbit and human anti-AMA1 antibodies have also been shown to efficiently inhibit parasite invasion of erythrocytes in vitro [12].

Sequencing of P. falciparum AMA1 from laboratory and field strains has produced over 130 non-redundant AMA1 sequences. These sequences result from an assortment of polymorphisms located throughout the molecule, but concentrated in domain I. The population distribution of these polymorphisms suggests that they have arisen due to diversifying selection, most likely to avoid the binding of inhibitory antibodies [13–15]. Consistent with this, protective responses induced by AMA1 have been shown to be strain-specific. Immunization of mice with recombinant P. chabaudi strain DS AMA1 conferred almost complete protection to homologous challenge, but little protection to challenge with the heterologous strain 556KA [9]. Similarly, in in vitro growth-inhibition studies, P. falciparum strain 3D7 was efficiently inhibited by polyclonal serum elicited by 3D7 AMA1, but the HB3 and W2mef strains were less efficiently inhibited by the same reagent [16]. Kennedy et al. showed that anti-AMA1 antibodies raised in rabbits inhibited merozoite invasion by heterologous parasite strains, but there was an inverse correlation between the degree of inhibition and the mutational distance of the strains studied [17]. Thus, inhibitory anti-AMA1 antibodies appear to recognize both polymorphic and conserved epitopes. AMA1 is currently being tested in several early clinical trials, and in one of these trials a combination of 3D7 and FVO AMA1 is being assessed in an attempt to overcome the problem of polymorphisms in this antigen [18,19].

Although AMA1 has been studied extensively, its biological function is still unknown. AMA1 is an unusual malaria vaccine candidate in that it is required for both merozoite invasion of erythrocytes [20] and sporozoite invasion of hepatocytes [21]. AMA1 is targeted to the micronemes of developing merozoites and is initially expressed as an 83 kDa precursor protein [22]. N-terminal processing produces a 66 kDa product that is released onto the surface of the free merozoite [23,24]. At the time of invasion, AMA1 is cleaved by a membrane-bound subtilisin-like protease, PfSUB2, resulting in the shedding of a 48 kDa fragment such that only the cytoplasmic, transmembrane, and a 29 residue membrane-adjacent fragment can be detected in ring-stage parasites [25,26]. The importance of the shedding process is not clear, but growth inhibitory anti-AMA1 polyclonal sera interfere with AMA1 shedding such that aberrantly processed forms of AMA1 are detected [26,27]. AMA1 has been shown not to contribute to the primary weak interaction between the merozoite and erythrocyte, but it is involved in secondary adhesion events which are thought to lead to tight junction formation immediately prior to host cell invasion [28,29]. Moreover, Toxoplasma gondii AMA1 forms a complex with proteins, including TgRON4, associated with the tight or moving junction that propels the parasite into the host cell [30], and P. falciparum AMA1 has recently been shown to interact with PfRON4 [31]. The effect of the immune response on this interaction is unknown.

Crystal structures of AMA1 have revealed that the antigen contains a pair of closely associated PAN domains [32,33]. Seven loops extend from the PAN scaffold and surround a long hydrophobic trough [33], which we have speculated is a ligand-binding pocket. Orthologs of AMA1 have been identified in all apicomplexan parasites, with the hydrophobic trough conserved across the phylum, including in T. gondii [34]. Therefore, the evolutionary acquisition of the loops onto the PAN scaffold that gave rise to the hydrophobic trough resulted from ancient events that preceded apicomplexan parasite divergence. In more recent times, *Plasmodium* species have incorporated numerous polymorphisms into some of these loops. The most highly polymorphic region of AMA1 surrounds one end of the hydrophobic trough in domain I, but dimorphic residues extend down one side of the protein surface into domains II and III [33,35]. This suggests that the hydrophobic trough is a major target of protective antibodies, but it is clear that epitopes in other regions of AMA1 are also recognized by inhibitory antibodies [27,36,37].

Given that AMA1 is in clinical trials, it is highly desirable to know more about the antigenic characteristics of the protein. In particular, information regarding the number and location of epitopes recognized by inhibitory monoclonal antibodies will facilitate vaccine development. Two growth-inhibitory monoclonal antibodies (mAbs) have been characterized: 1F9, which recognizes a polymorphic epitope on domain I [38,39], and 4G2, which recognizes a conserved region of domain II [40]. Mutagenesis studies indicate that their respective epitopes are located on loops close to but at opposite ends of the hydrophobic trough [39,40]. Here, by determining the crystal structure of the complex, we provide a detailed picture of the 1F9–AMA1 interaction. 1F9 has a very large footprint on AMA1, which includes hydrophobic trough residues as well as residues from the surrounding loops. We also show that mutagenesis of key residues on the loops surrounding the hydrophobic trough is sufficient to interfere with mAb 1F9 binding, and that the binding of both 1F9 and 4G2 to AMA1 is competed by naturally acquired human antibodies. These observations provide evidence in support of the hypothesis that the hydrophobic trough is an AMA1 ligand-binding site and a major target of protective immunity.

## Results

### mAb 1F9 Interacts with the AMA1 Hydrophobic Trough

**Figure 1:**
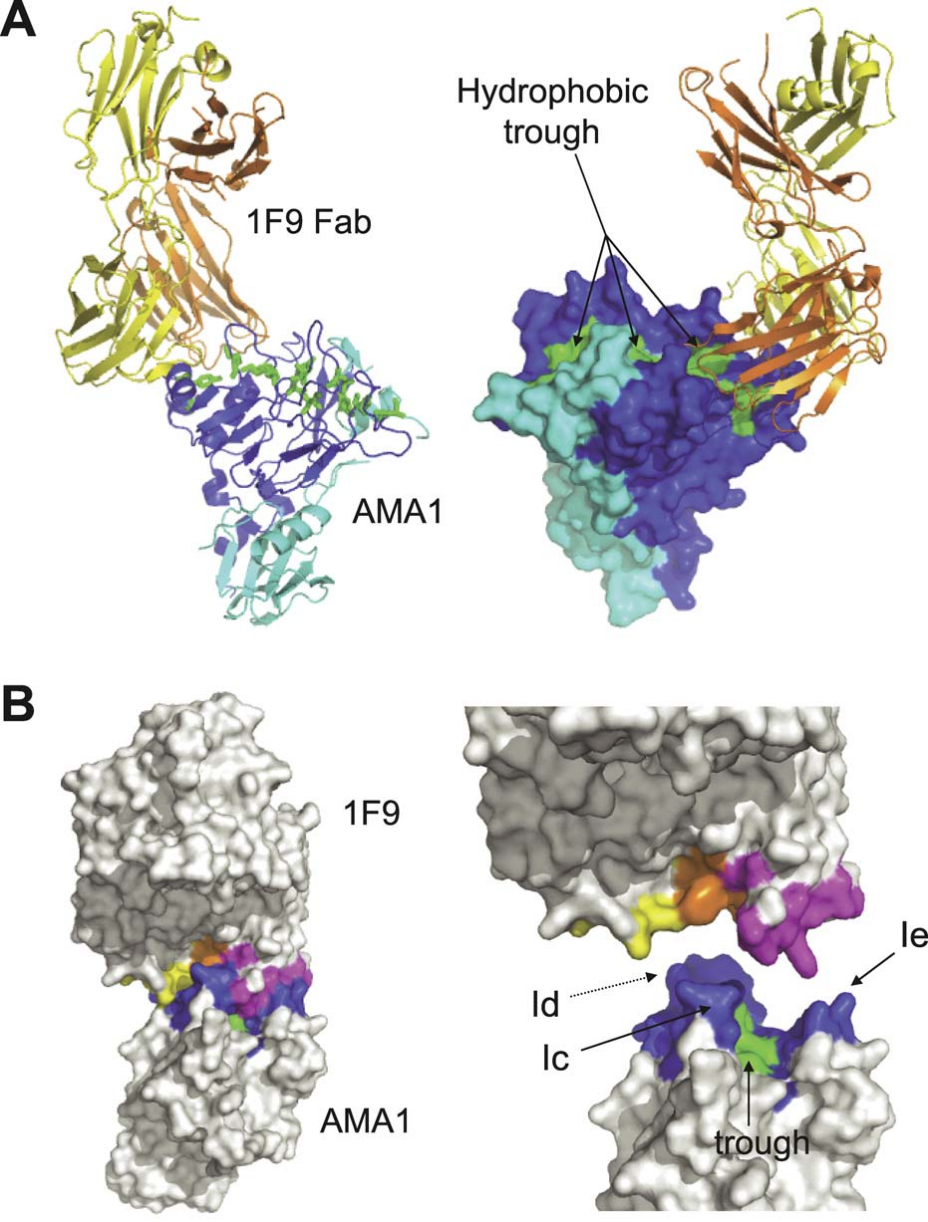
Structure of AMA1 in Complex with 1F9 Fab (A) Alternate views of AMA1 in complex with 1F9 Fab. Backbone trace of 1F9 Fab with the heavy chain coloured orange and the light chain coloured yellow. Backbone trace of AMA1 (left) or surface view (right) with domain I coloured dark blue and domain II coloured light blue. The AMA1 hydrophobic trough is coloured green. This figure and other figures depicting the structure were generated using PyMOL [56]. (B) Surface representation of the 1F9 Fab bound to AMA1. View of the complex (left), or 1F9 and AMA1 separated (right). Light chain residues that contact AMA1 (within 4 Å) are coloured yellow. Heavy chain residues that contact AMA1 are coloured orange (residues from the CDR loops) or pink (framework residues). AMA1 residues within 4 Å of 1F9 are coloured dark blue, with the hydrophobic trough residues contacting 1F9 coloured green. AMA1 loops Ic, Id, and Ie are indicated.

AMA1 domains I+II in complex with 1F9 Fab crystallized under identical conditions into two crystal forms: crystal form 1 and crystal form 2, which have been solved to 2.4 and 2.3 Å, respectively (Table 1). An overview of crystal form 1, the more complete of the two crystal structures, is shown in Figure 1A. 1F9 interacts exclusively with domain I of AMA1. The area of interaction encompasses one end of the group of solvent exposed hydrophobic residues that form part of the hydrophobic trough (coloured green in Figure 1A), which may be a ligand-binding site.

**Table 1:**
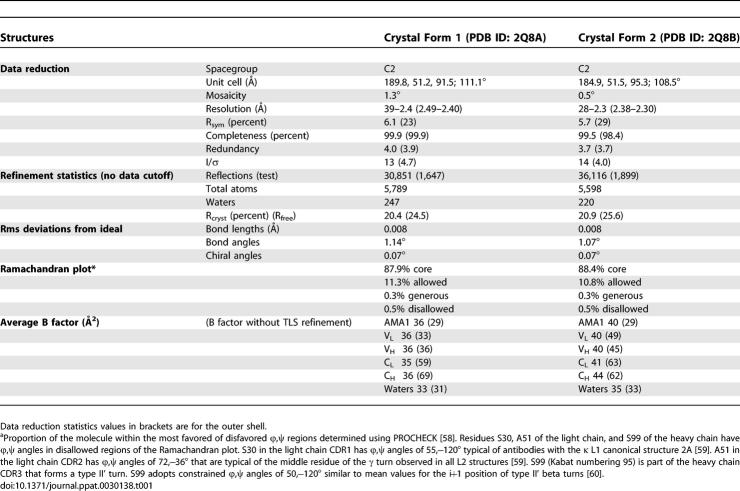
Crystallographic Data and Refinement Statistics

The total buried surface area at the 1F9–AMA1 interface is 2,470 Å^2^, with 1,250 Å^2^ buried on the AMA1 surface and 1,220 Å^2^ buried on the 1F9 surface. This is considerably larger than previously reported Fab–antigen buried surfaces, which typically range from 600 to 900 Å^2^. CDR loops of both the heavy and light chains of 1F9 interact with AMA1 (Figure 1A and 1B). In addition to the CDR loops, 1F9 contacts AMA1 using a large area of framework residues, which contact the hydrophobic trough (Figure 1B). There is, therefore, an interesting reciprocity to the structure in that the less variable framework ridge interacts with the conserved hydrophobic trough, and the variable antibody CDR loops interact with the polymorphic loops that surround the trough.

### The Principal 1F9 Contact Residues in AMA1 Are Polymorphic

**Figure 2:**
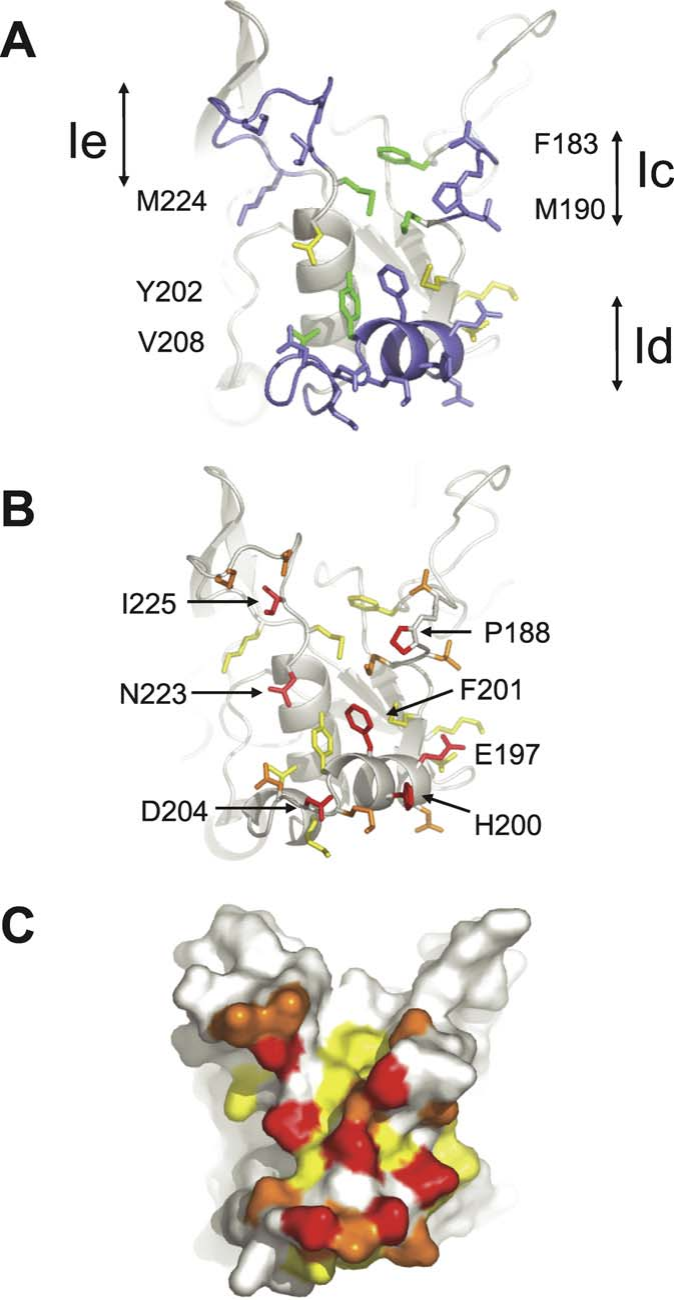
1F9 Epitope on AMA1 (A,B) Show identical views of the epitope with side chains that interact with AMA1 with a buried surface area of 7 Å^2^ or more shown in stick form. (C) Is the same view but showing the surface of the epitope. (A) Hydrophobic trough residues that contact 1F9 are shown in green with their residue numbers on either side of the figure. Loops Ic, Id, and Ie and their 1F9-contacting residues are shown in blue. Side chains that extend from the PAN domain scaffold and interact with 1F9 are coloured yellow. (B,C) Principal contact residues. Side chains contacting 1F9 with a surface area of greater than 78 Å^2^ or more are coloured red. Side chains with a contact surface area of between 68 and 38 Å^2^ are coloured orange. Side chains with a contact surface area of between 24 and 7 Å^2^ are coloured yellow. Surface area interactions were calculated using AREAIMOL [54].

1F9 covers one-half of the hydrophobic trough and surrounding loops on the surface of AMA1 (Figure 2A). Three of the domain I loops (Ic, Id, and Ie) comprise ∼90% of the buried surface on AMA1. The largest interaction is made by loop Id, which contributes 48% of the 1F9-covered area. The four residues that make the largest interactions, E197, H200, F201, and D204, each contributing ∼100 Å^2^, are all in the Id loop (Figure 2B and 2C). All four of these residues are polymorphic; residues 197 and 200 are highly polymorphic, residue 201 is less polymorphic, and residue 204 is strictly dimorphic (Table S1). Other loop residues that make a significant contribution to the 1F9 interface (∼80 Å^2^) are dimorphic residue 225 and nonpolymorphic P188 (Figure 2B and 2C).

### Mutations at Polymorphic Sites Abrogate 1F9 Binding

**Figure 3:**
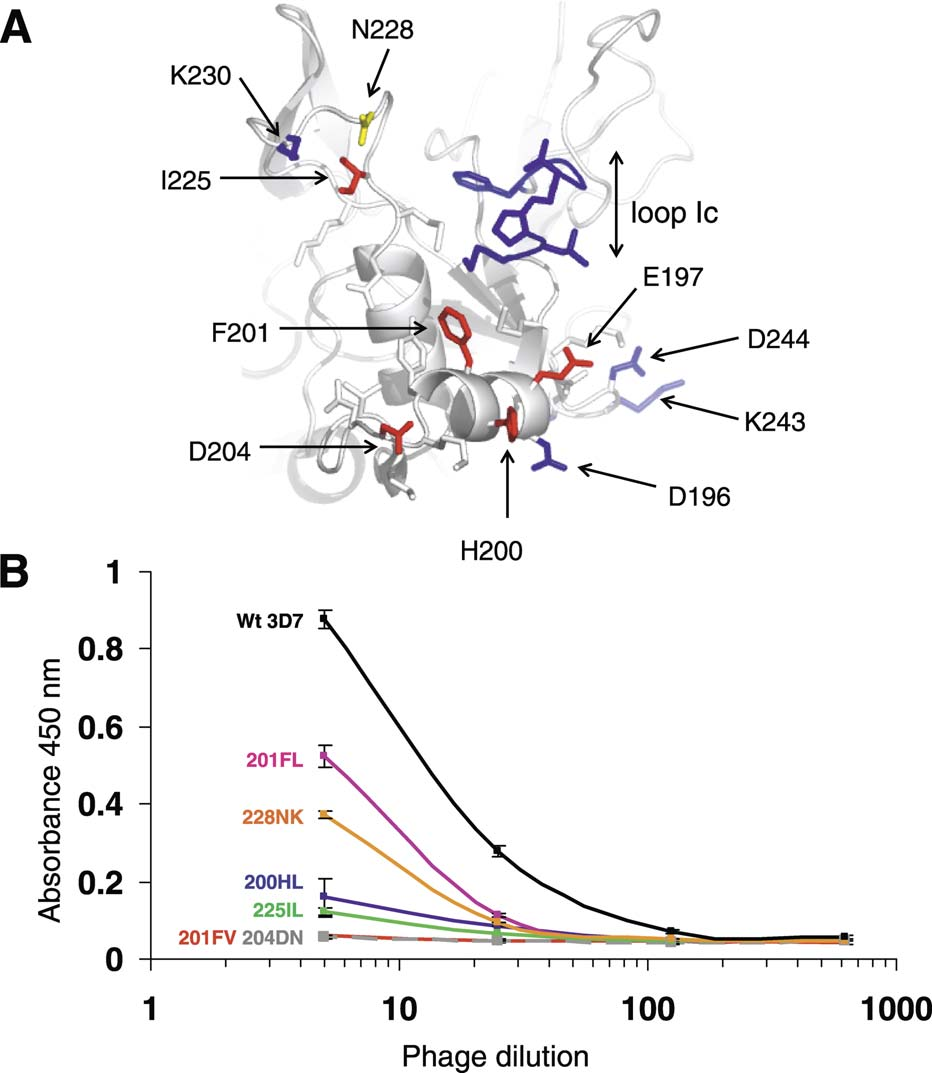
Effect of AMA1 Mutations on 1F9 Binding (A) Mapping of point and deletion mutations onto the AMA1 structure. Point mutations at positions 197, 200, 201, 204, and 225 (coloured red) disrupted 1F9 binding. Point mutations at position 228 (yellow) were partially disruptive. Point mutations at positions 196, 230, 243, and 244 (blue) had no effect on 1F9 binding. N-terminal deletion of loop Ic (blue) had no effect on binding [38]. (B) M13 phage expressing point mutations in domain I AMA1 were added to immobilized 1F9 at a series of dilutions. Bound phage were assayed by the addition to peroxidase-conjugated anti-M13 mAb followed by a colourimetric assay. Assays were carried out in duplicate and the error bars indicate the two measured absorbance values.

The structure of the 1F9–AMA1 complex is consistent with point mutants studied previously. Any substitution of E197 abrogated 1F9 binding, whereas mutating several other polymorphic sites (196, 230, 243, or 244) had no effect on 1F9 binding [39]. The crystal structure shows that residues 243 and 244 do not contact 1F9, whereas 196 and 230 are at the periphery of the interface where they are solvent-exposed such that mutations are accommodated (Figure 3A). A further series of point mutations have been generated in AMA1 domain I expressed on phage. Mutations were introduced at residues 200, 201, and 204 within loop Id, and residues 225 and 228 within loop Ie; all are sites of frequently occurring polymorphisms. The residues in 3D7 AMA1 were substituted with residues occurring in other AMA1 alleles, except for residue 225, where a conservative I-L mutation was analysed. Mutations at residues 200, 204, and 225 all abrogated binding (Figure 3). Substitution of valine for phenylalanine at position 201 also abrogated binding, whereas the substitution by leucine at this position only partially abrogated binding. Similarly, substituting lysine for aspartic acid at position 228 only partially abrogated binding (Figure 3). Deletion of residues in loop Ic had no effect on 1F9 binding [38]. This result was surprising because the deleted residues constitute 19% of the AMA1 interaction area. Presumably, because this area of interaction is at the periphery of the interface, deletions here can be accommodated. Overall, the mutational data highlighted the importance of polymorphic residues 197, 200, 201, 204, and 225, and these data were consistent with the crystal structures in that these residues all present large 1F9-interacting areas.

### Molecular Details of the AMA1-1F9 Interface

**Figure 4:**
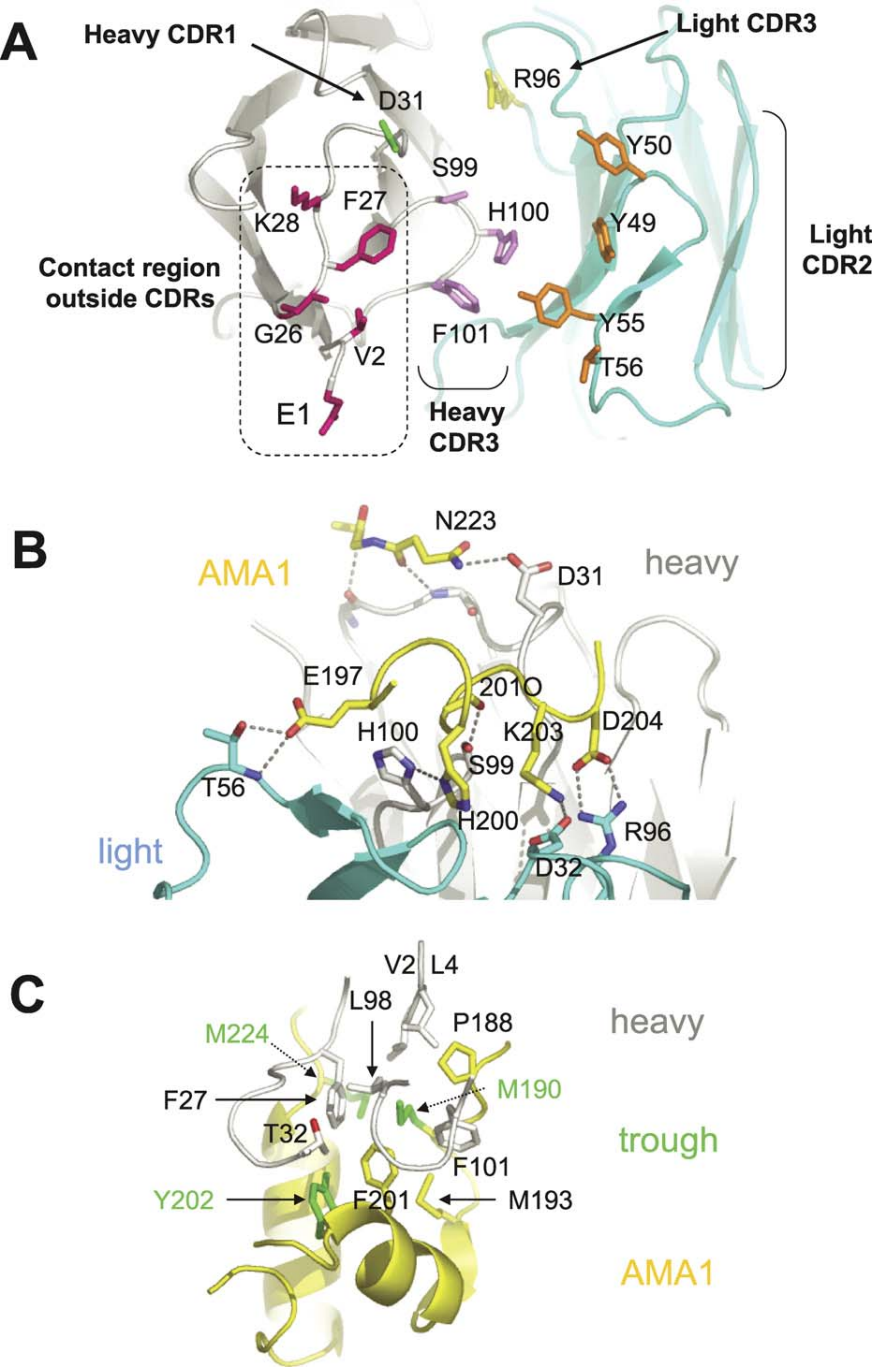
1F9 Recognition Surface and Details of the 1F9–AMA1 Interaction In all panels AMA1 main chain is coloured yellow, 1F9 light chain is light blue, and 1F9 heavy chain is grey/white. (A) View of the CDR loops on 1F9. Residues shown are those with an AMA1 contact surface area of at least 35 Å^2^. Light chain CDR2 and 3 side chains are coloured orange and yellow, respectively, with heavy chain CDR 1 and 3 side chains coloured green and violet, repectively. Residues coloured pink are in the antibody framework region. Amino acid numbering refers to the 1F9 sequence. In the Kabat numbering scheme [57] heavy chain residue S99 is S95, H100 is H96, and F101 is F102. Light chain numbering is the same as in the Kabat numbering scheme. (B) Principal hydrogen bond interactions between AMA1 and 1F9. Side view of the AMA1-1F9 interface showing that most hydrogen bond interactions occur between AMA1 loop Id (residues 197–204), 1F9 light chain residues and the heavy chain CDR3 (residues S99 and H100). 201O is the main-chain carbonyl oxygen of residue 201. (C) View of the 1F9 heavy chain hydrophobic residues sitting in the hydrophobic trough. Hydrophobic trough side chains are coloured green.

The principal residues on 1F9 that interact with AMA1 are derived from light chain CDRs 2 and 3, heavy chain CDR3, and heavy chain FR1 framework residues, with a minor contribution from heavy chain CDR1 (Figure 4A). The 1F9 heavy chain CDR3 loop is very short and consists of three residues (S99, H100, and F101, which form part of a tight type II' beta turn), all of which contact AMA1. Heavy chain FR1 framework residues E1 and V2, and G26, F27, and K28, form a cluster that, together with neighbouring framework residues, forms a contact surface area of 490 Å^2^ (Figure 4A). This accounts for >50% of the heavy chain interacting surface, and approximately 40% of the total 1F9 contact area. AMA1 therefore makes contact with a large region on the antibody surface outside the CDRs that is conserved in most human and most mouse antibodies.

One half of the 1F9–AMA1 interface is largely polar, whereas the other half of the interface is hydrophobic. Hydrogen bond interactions observed in both crystal forms are shown in Figure 4B. Most hydrogen bonds extend from residues on loop Id. For example, E197 forms hydrogen bonds with T56 in the light chain CDR2, H200 forms hydrogen bonds with H100 in the heavy chain CDR3, and D204 forms a salt bridge with R96 in the light chain CDR3. Mutations at these three sites (197, 200, 204) disrupted 1F9–AMA1 binding (Figure 3B), consistent with the importance of these hydrogen bond interactions at the interface. AMA1 residue N223, which extends from the domain I PAN helix, hydrogen bonds to D31 in the heavy chain CDR1.

The other half of the AMA1-1F9 interface consists of a large cluster of hydrophobic side chains (Figure 4C). 1F9 residues in the hydrophobic half of the interface are all part of the heavy chain. Heavy chain framework residues V2, L4, and F27 contribute 71, 16, and 86 Å^2^, respectively (total 173 Å^2^), to the interface. Heavy chain residues T32, L98, and F101 extend from antibody variable regions and contribute 24, 10, and 80 Å^2^, respectively (total 104 Å^2^), to the AMA1 interface. Thus, although conserved framework residues make the largest contribution to the hydrophobic cluster, variable residues, in particular F101 in heavy chain CDR3, also contribute.

**Figure 5:**
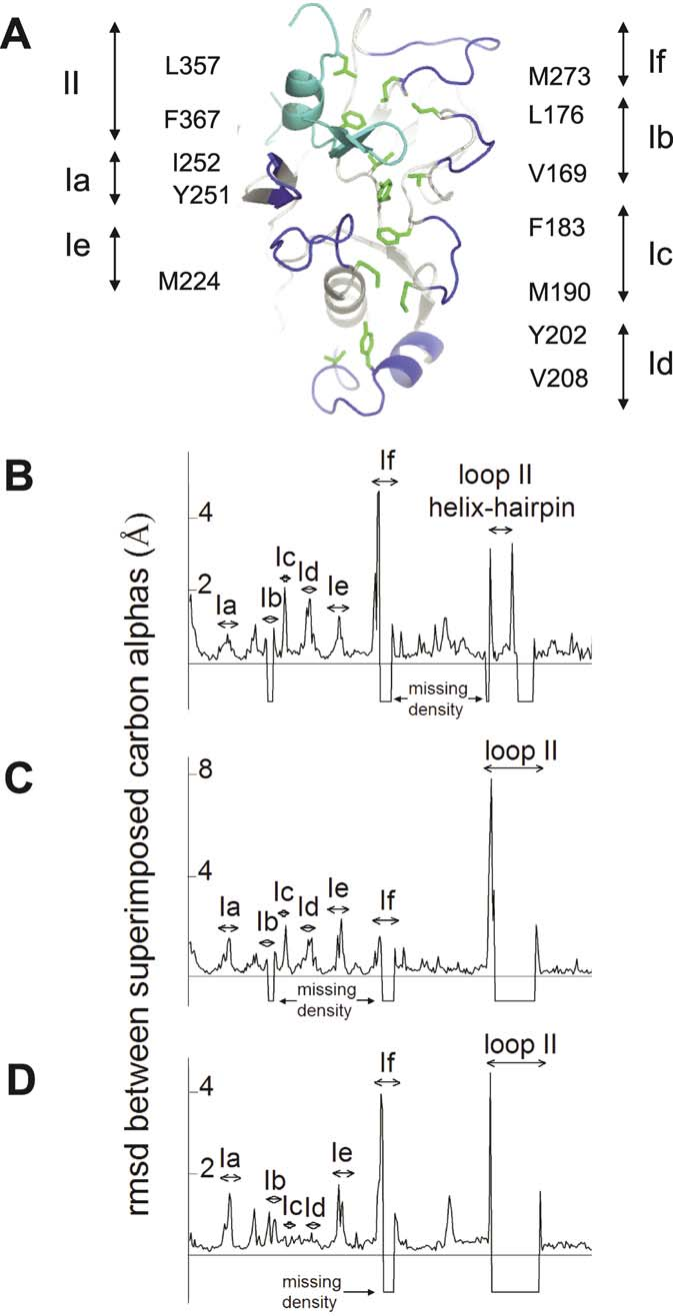
Comparison of Free and 1F9-Complexed AMA1 Structures (A) Structure of the hydrophobic trough and surrounding loops. Domain I loops, Ia-If, are shown in dark blue. The domain II loop, II, is shown in light blue. The 12 hydrophobic trough residues are shown in green with the label on either side of the figure showing the amino acid and numbering. I252 and Y251 are not adjacent to loop Ia, but other trough residue labels are shown next to the loop they are associated with. (B–D) Comparison of the main-chain trajectories of AMA1 structures.rmsds between superimposed carbon alphas were calculated using the program LSQKAB [54] and plotted for each residue. (A) Comparison between AMA1 model 1Z40 and AMA1-1F9 crystal form 1. (B) Comparison between AMA1 model 1Z40 and AMA1-1F9 crystal form 2. (C) Comparison between AMA1-1F9 crystal forms 1 and 2. In instances where residues were missing in either of the compared models the rmsd was assigned a value of −1.

AMA1 residues that contribute to the hydrophobic interface cluster include M224, M190, and Y202 from the hydrophobic trough (green, Figures 4C and 5A), P188 from loop Ic, M193 extending from the central PAN beta sheet, and F201 in loop Id. F201 contributes the largest surface area to the 1F9 interaction (101 Å^2^), and P188 also has a large interaction (87 Å^2^). Hydrophobic trough residues, M190, Y202, and M224 present smaller surface areas to 1F9 of 40, 24, and 24 Å^2^, respectively, whereas M193 plays a minor role, presenting a 12 Å^2^ surface to 1F9. Consistent with its position at the centre of the hydrophobic cluster, mutations at polymorphic residue 201, were critical for 1F9 binding. Mutating residue 201 to leucine, 201FL, partially reduced binding, whereas a smaller subsitution to valine, 201FV, totally disrupted binding (Figure 3B).

### Detailed Description of the Hydrophobic Trough

All previously determined structures of AMA1 are incomplete, particularly in the loops that surround the hydrophobic trough. The structure of AMA1-1F9 crystal form 1 is the only AMA1 structure describing loop If. In addition, the region of loop II adjacent to the hydrophobic trough is visible, making this the first AMA1 structure that provides a complete view of the hydrophobic trough (Figure 5A). Previously, nine hydrophobic trough residues were defined on the basis of sequence and surface exposure in the 1Z40 structure [33]. In crystal form 1, three additional residues have been identified as components of the trough. The 12 residues are hydrophobic in all *Plasmodium* AMA1 sequences, have hydrophobic side chains solvent exposed by 7 Å^2^ or more, and form part of a continuous surface on AMA1. The 12 hydrophobic residues are V169 and L176 on either side of loop Ib, F183 and M190 either side of loop Ic, Y202 and V208 within loop Id, M224 N-terminal to loop Ie, Y251 and I252 at the centre of the trough that extends from a loop following PAN beta strand 4 in domain I, M273 C-terminal side to loop If, and L357 and F367 within loop II (Figure 5A).

### 1F9 Binding Does Not Significantly Affect the AMA1 Structure

A plot of the distance between alpha carbons in the overlaid AMA1 structures 1Z40 (AMA1 alone) and AMA1-1F9 crystal form 1 reveals that the overall trajectory of the AMA1 backbone in the two structures is very similar, with root mean square deviations (rmsds) of less than 1 Å (Figure 5B). The regions where the rmsd exceeds 1 Å all correspond to the loops surrounding the hydrophobic trough. The deviations of loops Ia, Ic, Id, and Ie are not large and rmsds do not exceed 2 Å.

The largest variations in the main-chain conformations of AMA1 among the 1Z40 and AMA1-1F9 crystal form 1 and 2 structures were observed in loop If and loop II. Loops If and II were largely invisible in AMA1-1F9 crystal form 2, but where main-chain density was observed, the loop trajectories differed by 4–8 Å (Figure 5C and 5D). In the 1Z40 structure, loop II extended up domain I to form a helix and beta-hairpin adjacent to the hydrophobic trough. Although sections of loop II were invisible in 1F9–AMA1 crystal form 1, the conformation of the helix-beta-hairpin was identical to that observed in the 1Z40 structure, with rmsds not exceeding 1 Å. Therefore, although loop II can form multiple conformations, when the loop II helix and beta-hairpin is observed it forms a precise conformation that packs against domain I to form part of the hydrophobic trough. Loops Ic and Id have identical conformations in crystal forms 1 and 2 (Figure 5D), presumably because these loops form a major part of the AMA1-1F9 interface. In contrast, within loop Ie, residue 225 interacts with 1F9 in a consistent manner, whereas the remainder of the loop adopts a different conformation in the two crystal forms.

### Antibodies from Malaria-Infected Individuals Recognize the 1F9 Epitope

**Figure 6:**
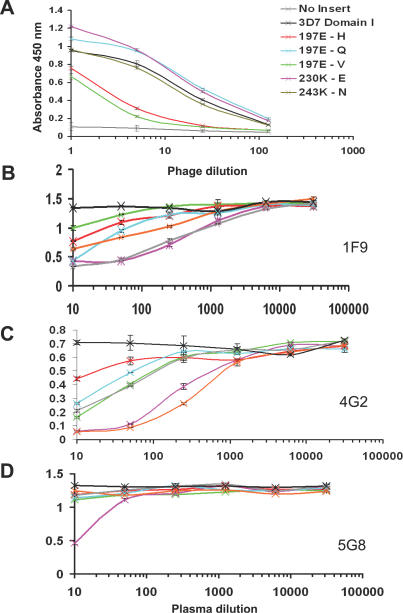
Binding of Human Plasma to AMA1 (A) Point mutations abrogate binding of AMA1 domain I to human antibodies. Phage expressing AMA1 domain I were tested for their ability to bind to immobilised AMA1-affinty purified human antibodies from a pool of Papua New Guinean blood donors. Control (black) is M13 phage expressing strain 3D7 AMA1 domain I. Negative control (grey) is phage expressing no insert. Point mutations tested were: residue 197E changed to (H red, Q cyan, V green), or residue 230 K-E (violet), or residue 243 K-N. Assays were carried out in duplicate and the error bars indicate the two measured absorbance values. (B–D) Individual human plasma compete for 1F9 binding to AMA1. Full-length 3D7 AMA1 ectodomain was immobilised on plastic. MAbs 1F9 (A), 4G2 (B), and 5G8 (C) were allowed to bind and the ability of a set of human plasma to compete for mAb binding was tested: plasma P8 (green), plasma P45 (pink), plasma P60 (red), plasma P69 (light blue), plasma P111 (orange), and plasma M157 (grey). Pooled plasma derived from a malaria unexposed individuals served as a negative control (black). Assays were carried out in duplicate and error bars indicate the two measured absorbance values.

Polymorphism-scanning mutagenesis and competition ELISA approaches were used to determine whether the human immune response to P. falciparum parasites involves the production of antibodies that resemble 1F9 in their binding properties. 3D7 AMA1–purified antibodies were adsorbed onto plastic and M13 phage expressing 3D7 domain I were allowed to bind. The conservative substitution 197E-Q, which had previously been shown to reduce but not ablate 1F9 binding [39], had no discernable effect on the binding of polyclonal human antibodies (Figure 6A). In contrast, more radical substitutions (197E-V and 197E-H) dramatically reduced AMA1 binding to 1F9 and to human antibodies. E197 hydrogen bonds with T56 in the 1F9 light chain (see Figure 4B) and the E-Q mutation could preserve these hydrogen bonds. However, these hydrogen bonds would not be preserved with valine or histidine substituting for E197. Also, because of the hydrophobicity of valine and the size and charge of histidine, these mutations may cause significant pertubations of the surface topology in this region of AMA1. Mutating polymorphic residues 230 or 243, at either side of the 1F9 epitope (see Figure 3A), had little effect on the binding of human antibodies to AMA1 domain I (Figure 6A). The parallels between the binding of human antibodies and 1F9 to various forms of AMA1 indicate that antibodies targeting the 1F9 epitope are a component of the human antibody response to AMA1.

In order to confirm the presence of 1F9-like antibodies in malaria-infected human plasma, the ability of individual human plasma to compete with 1F9 for AMA1 binding was also tested. Plasma (from Papua New Guinean blood donors) were pre-screened for their ability to recognize recombinant full-length 3D7 AMA1 ectodomain [12]. Plasma that reacted strongly with 3D7 AMA1 were tested for their ability to compete for AMA1 binding with 1F9 and with two other anti-AMA1 mAbs, 5G8 and 4G2. All six Papua New Guinean plasma samples reduced the binding of 1F9 to 3D7 AMA1 to some degree, whereas plasma from individuals with no exposure to malaria did not inhibit 1F9 binding (Figure 6B). The Papua New Guinean plasma samples also contained antibodies to the conserved epitope recognized by the inhibitory antibody 4G2. Only one of the six plasma contained antibodies to the epitope recognized by the non-inhibitory mAb 5G8. 5G8 recognizes a linear epitope at the N-terminus of AMA1, which is not present on the 66 kDa processed form of the antigen [22,38,39].

The reverse competition experiment, in which the binding of human antibodies to AMA1 was competed by excess 1F9, indicated that human antibodies recognizing the 1F9 epitope are relatively abundant (up to 40% of the total AMA1 reactivity) in the plasma of some individuals (Figure S1). This result is consistent with the AMA1 domain 1 mutagenesis experiment, suggesting that the 1F9 epitope is an important target of the human anti-AMA1 antibody response in individuals exposed to malaria.

## Discussion

The crystal structures described here show that mAb 1F9, which inhibits merozoite invasion, interacts with an epitope on AMA1 that includes residues in the hydrophobic trough and on loops surrounding the trough. This is consistent with the hypothesis that the hydrophobic trough is a ligand-binding pocket that plays an essential role in merozoite attachment and/or invasion. The residues presenting the largest surface area to 1F9 are located on the loops surrounding the hydrophobic trough. Most of these residues are polymorphic, and mutagenesis confirmed that they are critical to 1F9 binding, consistent with the parasite strain specificity observed for 1F9-mediated inhibition [39]. This contrasts with the epitope of the non-inhibitory mAb F8.12.19, which is located in domain III and conserved in several plasmodial species [41].

The 1F9–AMA1 interface is unusual in that the buried surface area is very large and antibody framework residues make extensive contact with the antigen. These framework residues form a ridge that protrudes into the hydrophobic trough, giving complementary surfaces unlike most antibody/protein interfaces, which are typically flat [42]. Exceptions include antibodies with long heavy chain CDR3 loops such as mAb b12, where the CDR3 protrudes into the CD4 binding site of HIV gp120 [43]. The 1F9 heavy chain CDR3 loop is very short and, as a result, recessed such that neighbouring framework residues are exposed. These residues from heavy chain FR1 are commonly found in mouse and human antibodies apart from residue 26, which appears to have resulted from an N to K somatic mutation (Figure S2). The ridge framework residues, together with a short CDR3 loop, may allow antibodies to interact with antigens possessing clefts. Consistent with this, framework residues of the neutralizing mAb 17-IA make important contacts with a receptor-binding canyon on human rhinovirus 14 [44].

Serological studies indicate that a significant proportion of the human antibody response to AMA1 is directed towards polymorphic epitopes [45,46]. The 1F9 epitope encompasses the most polymorphic surface region of AMA1, and human antibodies compete with 1F9 for binding to full-length recombinant AMA1. These observations suggest that that the region containing the 1F9 epitope may be an antigenic hot-spot. Residue 197, on loop Id, is the most polymorphic site in AMA1 and appears to be a critical residue in this dominant epitope, as mutation of this residue not only ablates 1F9 binding, but also markedly reduces the binding of human antibodies. In an initial attempt to quantify the antigenicity of the 1F9 epitope, the ability of 1F9 to compete for human plasma was tested. These preliminary experiments suggest that in some, but not all, individuals, a significant fraction of anti-AMA1 antibodies bind to the 1F9 epitope region (Figure S1).

It is not suprising that the unusual AMA1 loops are targets of the antibody binding given their surface exposure and flexibility [47]. The loops surrounding the hydrophobic trough in the region of the 1F9 epitope are moderately flexible, whereas loops II and If are considerably more flexible. It would be anticipated that antibodies binding to the region of the trough surrounded by the more flexible loops would be parasite-growth inhibitory, and, indeed, inhibitory mAb 4G2 is known to bind to loop II [40]. In contrast to the loops contributing to the 1F9 epitope, loops II and If contain very few polymorphisms. Loop II has no polymorphisms and loop If only has two polymorphic sites: 267, with conservative substitutions (glutamate or glutamine), and residue 273, which is mostly lysine with isoleucine occurring at a low frequency. Thus, there is a striking difference in the abundance of polymorphisms in the various loops surrounding the hydrophobic trough, and this is inversely correlated with loop flexibility. It is not clear why the more flexible loops are less polymorphic, but they border the non-polymorphic face of AMA1, and this face may be partially hidden on the parasite surface. Consistent with this, 4G2 is known to be a more effective inhibitor as the Fab fragment [27]. Flexibility itself may offer partial antigenic “protection,” or there may be other fitness constraints that prevent mutations within the flexible loops.

Antibodies to AMA1 could inhibit merozoite invasion by several mechanisms. For example, it has been reported that inhibitory antibodies block AMA1 processing events [27]. On the basis of the information presented here, a reasonable conclusion is that 1F9 inhibits merozoite invasion by blocking the access of a ligand to the hydrophobic trough. Although the 4G2 epitope has been mapped to the opposite face of AMA1, its epitope also lies adjacent to the hydrophobic trough. It is therefore likely that these different monoclonal antibodies inhibit merozoite invasion through a common mechanism.

A hydrophobic trough-binding ligand has not been identified, but AMA1 is known to be associated with a rhoptry neck protein RON4 that is part of the moving junction between the surface of the invading merozoite and the erythrocyte membrane [30,31]. The nature of the interaction between RON4 and AMA1 is not understood; however, if the hydrophobic trough is involved in binding, it is unclear how antibodies would interfere with the AMA1/RON4 association if surface exposure of the complex is limited to the moving junction.

The polymorphic nature of merozoite surface proteins is a major problem confronting the development of a malaria vaccine incorporating one or more of these antigens. In a vaccine trial in Papua New Guinea, a three-component vaccine containing one form of MSP2 reduced parasite densities, but the effect was restricted to parasites expressing MSP2 genotypes of the same dimorphic form as the vaccine component [48]. AMA1 lacks the highly polymorphic repetitive sequences seen in MSP2, but close to 10% of the residues in the ectodomain are polymorphic. Consequently, large numbers of AMA1 haplotypes occur in endemic areas [13–15], and a vaccine incorporating a single allelic form of AMA1 is also likely to induce protection to only a subset of P. falciparum genotypes.

Given the number of alternative amino acid residues that are found in the 1F9-binding region, it seems unlikely that the strategy of combining two forms of AMA1 (3D7 and FVO) [19] will result in a vaccine that, acting alone, induces effective titres of antibodies to this polymorphic site on the majority of P. falciparum genotypes. In the context of endemic malaria, such a vaccine may still achieve a satisfactory level of efficacy because many infections are avirulent, and these have the potential to broaden the specificity of the immune response primed by the vaccine. These considerations underline the importance of analyzing the genotypes of breakthrough parasitemias in vaccine trials. If an AMA1 vaccine containing one or two forms of the antigen only protects against a minority of parasite genotypes, an alternative strategy may be to generate a form of AMA1 that targets the immune response to conserved regions of the molecule, including the “non-polymorphic” end of the hydrophobic trough. A potential strategy to achieve this may be to construct a form of AMA1 in which the polymorphic loops are removed or truncated.

## Materials and Methods

### Fab 1F9 production, sequencing, crystallization, and data collection.

AMA1 domains I+II were expressed in E. coli and refolded and purified using procedures described previously [49]. Mouse mAb 1F9 (isotype IgG2b with a Kappa light chain) was produced in hybridoma cell cultures and purified by protein G affinity chromatography. 10 mg of 1F9 was digested with 2.25 mg papain (Sigma) in 26 mM tris (pH 7.5), 4 mM EDTA, 4 mM 2-mercaptoethanol for 3 h at 20 °C. Proteolysis was terminated by adding 100 mM iodoacetic acid for 1 h at 4 °C. The 1F9 Fab fragment was purified by cation exchange chromatography (Mono S) in 20 mM acetic acid–NaOH (pH 5.0), eluting at 100 mM NaCl. The fragment was further purified by size exclusion chromatography (Hi Prep sephacryl S-200) in 20 mM acetic acid–NaOH (pH 5.0). The purified protein was dialysed into water, concentrated to 10 mg/ml, and stored at 4 °C in 0.02% sodium azide.

For crystallization, AMA1 domains I+II and 1F9 Fab were mixed (with the Fab in slight excess) and added to well solution (20 mM MES [pH 6.0], 10 mM MnCl_2_, 6% PEG 3350). Crystallization was carried by vapour diffusion in hanging drops and yielded two crystal forms that grew within 5 days. Both crystal forms were stabilized in 20 mM MES (pH 6.0), 10 mM MnCl_2_, 10% PEG 3350 for manipulations, and momentarily suspended in stabilization solution plus 25% methylpetanediol or glycerol for cryofreezing. The crystals were maintained at 100 K and data collected in-house using a rotating anode generator and image plate detector. 180° of data was collected in 0.5° sections and indexed using D*TREK [50].

1F9 heavy and light chain variable domain sequences were determined following the method of Gilliland et al. [51]. Sequences are shown alongside the closest matching mouse genomic sequences in Figures S2 and S3.

### Structure determination and refinement.

The structures were solved by molecular replacement with PHASER [52] using P. falciparum AMA1 structure 1Z40 [33] and mouse IgG2b/Kappa Fab structure 2CGR [53]. AMA1-1F9 complex structures were refined using REFMAC5 [54] and model building carried out using O [55]. The structures were divided into 5 TLS groups: AMA1, the light variable domain, the light constant domain, the heavy variable domain, and the heavy constant domain.

In crystal form 2, the domain II loop is disordered and AMA1 residues A355–A387 were not observed in the electron density. Similarly, the domain I loop, If, was disordered (AMA1 residues A264–A272). There was one gap in the heavy chain constant domain between residues H128 and H132. In crystal form 1, there were two gaps in the AMA1 structure. Both of these gaps were in the domain II loop: A351–A353 and A377–A389. In addition, there were two electron density gaps in the heavy chain constant domain: residues H128–H131 and H155–H159.

### AMA1 expression on phage, mutagenesis, and 1F9 binding.

AMA1 mutations were generated using the Kunkel method as described previously [39]. Mutated fragments were inserted into phagemid vector pHENH6 and AMA1-expressing phage were generated by inoculating transformants with M13KO7 helper phage. Phage preparations were normalized for AMA1 expression by performing ELISAs to examine the binding of mAb 9E10 to a C-terminal c-Myc epitope. 1F9 binding was examined by binding phage to immobilized 1F9 and detected using peroxidise conjugated anti-M13 mAb followed by colourimetric assay and measurement of light absorbance at 450 nm [39].

### Competition ELISAs.

100 μl of 0.3 μg/ml of full-length AMA1 ectodomain was immobilized on plastic. 100 μl dilutions of human plasma from Papua New Guinean blood donors (previously selected for reactivity to a series of malaria antigens, including full-length 3D7 AMA1 ectodomain) was added followed by 10 μl of 3.0 μg/ml mAb (either 1F9, 5G8, or 4G2). The ELISA plate was washed, peroxidase-conjugated anti-mouse antibodies added, and the assay developed. For reverse competition experiments, plasma was added prior to mAbs, and peroxidase-conjugated anti-human antibodies added to the washed plate.

## Supporting Information

Figure S1 1F9 Competes with Human Plasma for Binding to AMA1 Full-length 3D7 AMA1 ectodomain was immobilised on plastic and bound to individual human plasma: serum P8 (green), serum P45 (pink), serum P111 (orange), and serum M157 (grey).
(A) Ability of mAb 1F9 to compete for plasma was tested by adding 1F9 to AMA1 immediately prior to the addition of plasma.
(B) Competition experiment with mAb 4G2. The observed lack of competition does not necessarily indicate a low human antibody response to the 4G2 epitope region and may result from a low affinity of 4G2 for AMA1, consistent with the enhanced ability of human antibodies to compete for 4G2 (Figure 6C). The increased signal at high 4G2 concentrations may be due to cross-reactivity between 4G2 and the conjugated anti-human reagent. Alternatively, binding of 4G2 may induce conformational changes such that there is an increase in binding of human antibodies to neighbouring epitopes.
(1.4 MB PDF)

Figure S2 1F9 Heavy Chain Sequence The VH sequence is the closest matching mouse germline heavy chain variable gene (Genebank accession number X03571). D? is the short sequence, ctttccc, attributable to a D sequence, but showing no homology to any of the mouse D minigenes. 1F9 utlizes the mouse heavy chain J2 minigene (accession number X63166). CH1 sequence is part of the heavy chain gamma-2b C-region (accession number L00051). Somatic mutations are highlighted in pink. Sequences shown in blue are recombination recognition sequences and introns that are not present in the mRNA. 7 mer and 9 mer refer to the recombination recognition motifs that are capitalized. Variable antibody CDR sequences are underlined. Numbers in green indicate the area of interaction with AMA1 in crystal form 2. Underlined areas indicate a hydrogen bond interaction with AMA1. Arrows indicate stretches of beta strand and the cylinder an alpha helix.
(44 KB PPT)

Figure S3 1F9 Light Chain Sequence VL is the closest matching mouse variable kappa light chain gene, IgVk19–32 (accession number AJ235968). 1F9 light chain uses the kappa J2 minigene (accession number L80040). CL is part of the kappa light chain constant gene sequence (accession number V01569).
(40 KB PPT)

Table S1 Polymorphisms within the 1F9 Epitope Table of AMA1 residues that contact 1F9 showing the polymorphisms in the field, and the polymorphic difference probability (the probability that two sequences differ at a position). Table S1 also shows the 1F9-buried surface for each AMA1 residue in crystal form 2. Polymorphic residues tend to be more exposed and present a larger surface area to 1F9.
(43 KB DOC)

### Accession Numbers

The Protein Data Bank (http://www.rcsb.org/pdb/) ID numbers for the structures discussed in this paper are crystal form 1 (2Q8A) and crystal form 2 (2Q8B).

## Abbreviations

AMA1: apical membrane antigen 1
CDR: complementarity-determining region
mAb: monoclonal antibody
rmsd: root mean square deviation
