# Correction: Structure of the Malaria Antigen AMA1 in Complex with a Growth-Inhibitory Antibody

**DOI:** 10.1371/journal.ppat.0030172

**Published:** 2007-11-30

**Authors:** Andrew M Coley, Aditi Gupta, Vince J Murphy, Tao Bai, Hanna Kim, Michael Foley, Robin F Anders, Adrian H Batchelor

Correction for: Coley AM, Gupta A, Murphy VJ, Bai T, Kim H, et al. (2007) Structure of the malaria antigen AMA1 in complex with a growth-inhibitory antibody. PLoS Pathog 3(9): e138. doi: 10.1371/journal.ppat.0030138.

In *PLoS Pathogens,* volume 3, issue 9:

In the original version of this paper the author order was listed incorrectly. The proper order should read as above.

