# Supplementary material for: Structure of the Malaria Antigen AMA1 in Complex with a Growth-Inhibitory Antibody
Source: PLoS Pathog. 2007 Sep 28;3(9):e138. doi: 10.1371/journal.ppat.0030138 (PMC2323298; doi:10.1371/journal.ppat.0030138)
Supplement: Figure S2 — The VH sequence is the closest matching mouse germline heavy chain variable gene (Genebank accession number X03571). D? is the short sequence, ctttccc, attributable to a D sequence, but showing no homology to any of the mouse D minigenes. 1F9 utlizes the mouse heavy chain J2 minigene (accession number X63166). CH1 sequence is part of the heavy chain gamma-2b C-region (accession number L00051). Somatic mutations are highlighted in pink. Sequences shown in blue are recombination recognition sequences and introns that are not present in the mRNA. 7 mer and 9 mer refer to the recombination recognition motifs that are capitalized. Variable antibody CDR sequences are underlined. Numbers in green indicate the area of interaction with AMA1 in crystal form 2. Underlined areas indicate a hydrogen bond interaction with AMA1. Arrows indicate stretches of beta strand and the cylinder an alpha helix. (44 KB PPT) [file ppat.0030138.sg002.ppt]

## Slide 1
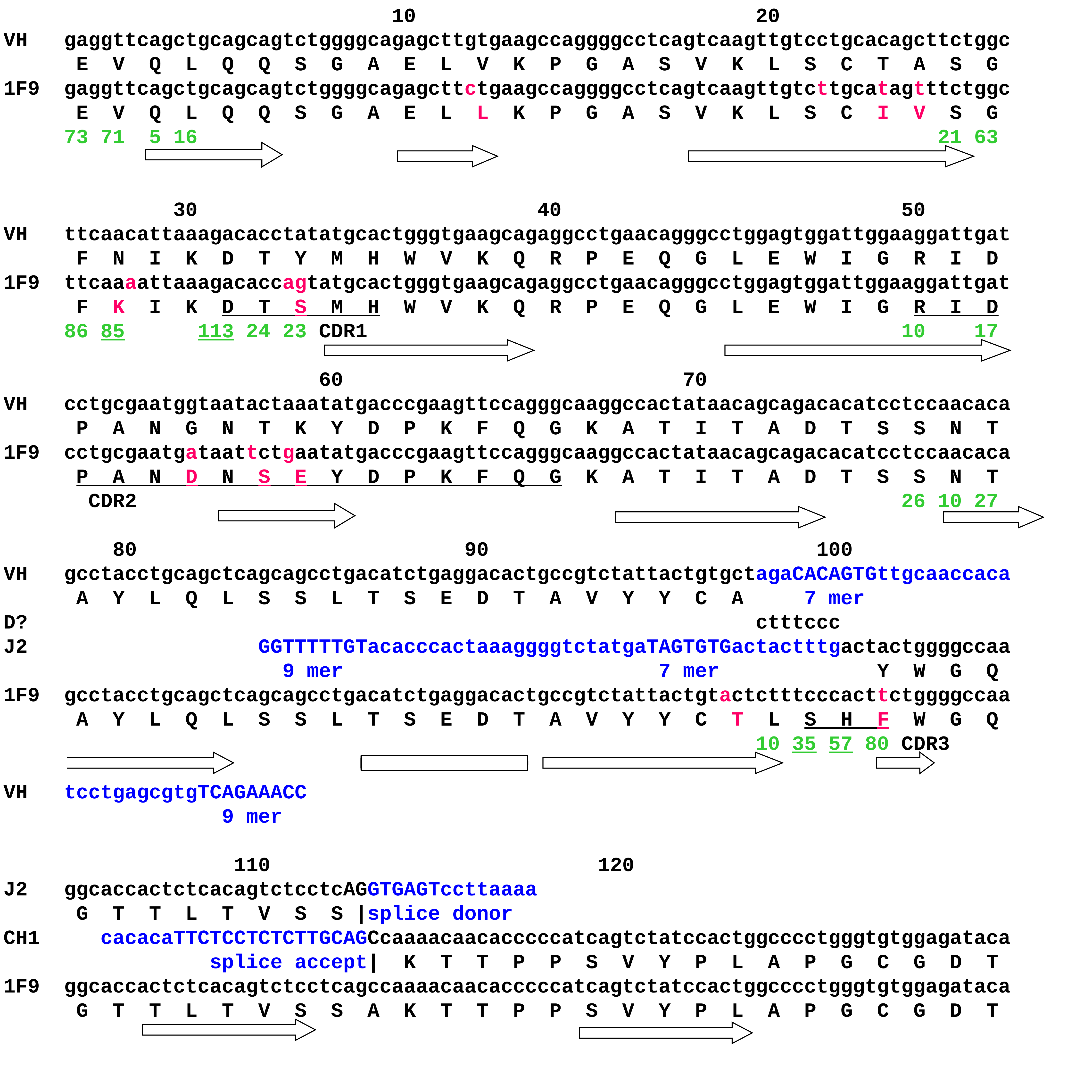

10 20
VH gaggttcagctgcagcagtctggggcagagcttgtgaagccaggggcctcagtcaagttgtcctgcacagcttctggc
 E V Q L Q Q S G A E L V K P G A S V K L S C T A S G
1F9 gaggttcagctgcagcagtctggggcagagcttctgaagccaggggcctcagtcaagttgtcttgcatagtttctggc
 E V Q L Q Q S G A E L L K P G A S V K L S C I V S G
 73 71 5 16 21 63
 30 40 50
VH ttcaacattaaagacacctatatgcactgggtgaagcagaggcctgaacagggcctggagtggattggaaggattgat
 F N I K D T Y M H W V K Q R P E Q G L E W I G R I D
1F9 ttcaaaattaaagacaccagtatgcactgggtgaagcagaggcctgaacagggcctggagtggattggaaggattgat
 F K I K D T S M H W V K Q R P E Q G L E W I G R I D
 86 85 113 24 23 CDR1 10 17
 60 70
VH cctgcgaatggtaatactaaatatgacccgaagttccagggcaaggccactataacagcagacacatcctccaacaca
 P A N G N T K Y D P K F Q G K A T I T A D T S S N T
1F9 cctgcgaatgataattctgaatatgacccgaagttccagggcaaggccactataacagcagacacatcctccaacaca
 P A N D N S E Y D P K F Q G K A T I T A D T S S N T
 CDR2 26 10 27
 80 90 100
VH gcctacctgcagctcagcagcctgacatctgaggacactgccgtctattactgtgctagaCACAGTGttgcaaccaca
 A Y L Q L S S L T S E D T A V Y Y C A 7 mer
D? ctttccc
J2 GGTTTTTGTacacccactaaaggggtctatgaTAGTGTGactactttgactactggggccaa
 9 mer 7 mer Y W G Q
1F9 gcctacctgcagctcagcagcctgacatctgaggacactgccgtctattactgtactctttcccacttctggggccaa
 A Y L Q L S S L T S E D T A V Y Y C T L S H F W G Q
 10 35 57 80 CDR3
VH tcctgagcgtgTCAGAAACC
 9 mer
 110 120
J2 ggcaccactctcacagtctcctcAGGTGAGTccttaaaa
 G T T L T V S S |splice donor
CH1 cacacaTTCTCCTCTCTTGCAGCcaaaacaacacccccatcagtctatccactggcccctgggtgtggagataca
 splice accept| K T T P P S V Y P L A P G C G D T
1F9 ggcaccactctcacagtctcctcagccaaaacaacacccccatcagtctatccactggcccctgggtgtggagataca
 G T T L T V S S A K T T P P S V Y P L A P G C G D T
