# Supplementary material for: Structure of the Malaria Antigen AMA1 in Complex with a Growth-Inhibitory Antibody
Source: PLoS Pathog. 2007 Sep 28;3(9):e138. doi: 10.1371/journal.ppat.0030138 (PMC2323298; doi:10.1371/journal.ppat.0030138)
Supplement: Figure S3 — VL is the closest matching mouse variable kappa light chain gene, IgVk19–32 (accession number AJ235968). 1F9 light chain uses the kappa J2 minigene (accession number L80040). CL is part of the kappa light chain constant gene sequence (accession number V01569). (40 KB PPT) [file ppat.0030138.sg003.ppt]

## Slide 1
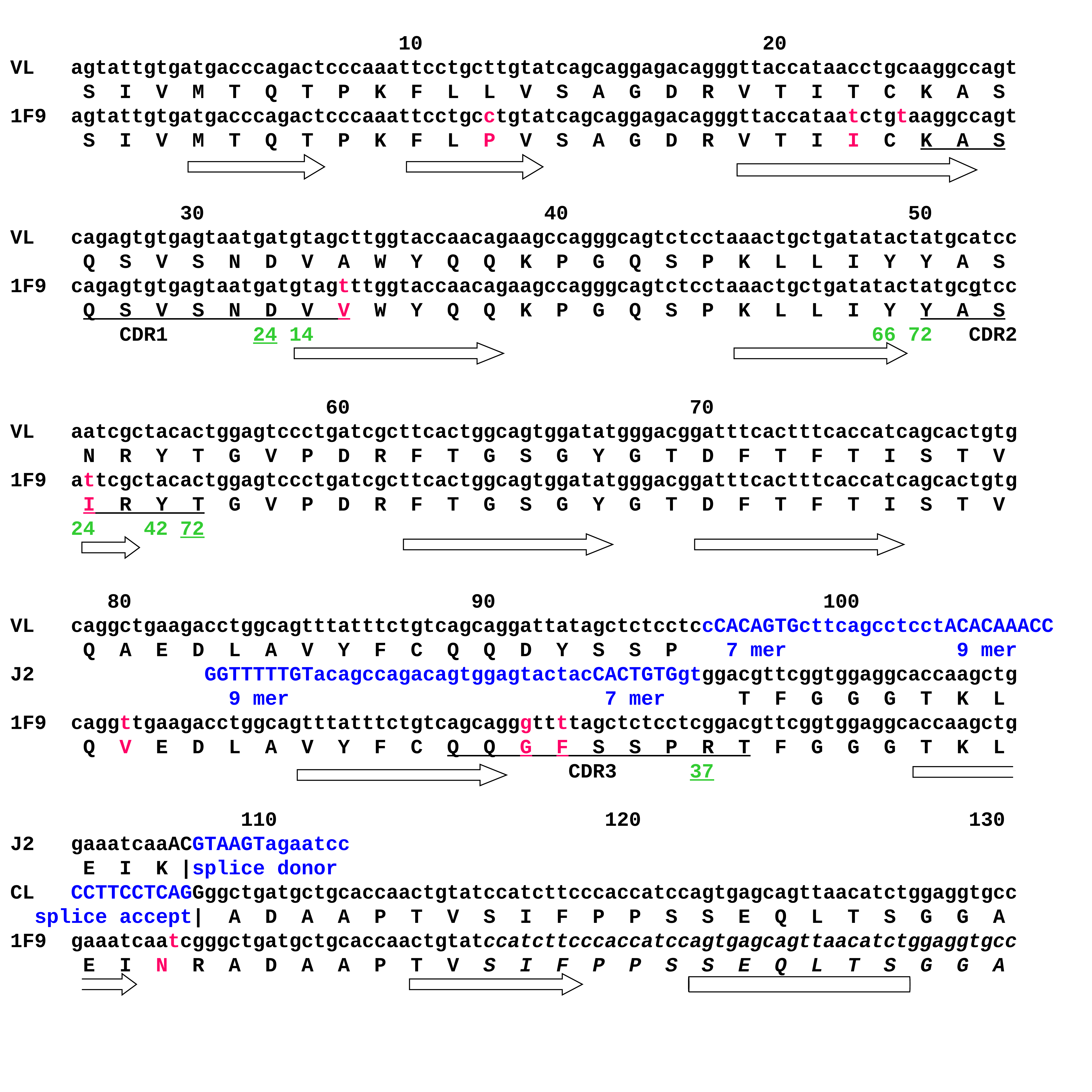

10 20
VL agtattgtgatgacccagactcccaaattcctgcttgtatcagcaggagacagggttaccataacctgcaaggccagt
 S I V M T Q T P K F L L V S A G D R V T I T C K A S
1F9 agtattgtgatgacccagactcccaaattcctgcctgtatcagcaggagacagggttaccataatctgtaaggccagt
 S I V M T Q T P K F L P V S A G D R V T I I C K A S
 30 40 50
VL cagagtgtgagtaatgatgtagcttggtaccaacagaagccagggcagtctcctaaactgctgatatactatgcatcc
 Q S V S N D V A W Y Q Q K P G Q S P K L L I Y Y A S
1F9 cagagtgtgagtaatgatgtagtttggtaccaacagaagccagggcagtctcctaaactgctgatatactatgcgtcc
 Q S V S N D V V W Y Q Q K P G Q S P K L L I Y Y A S
 CDR1 24 14 66 72 CDR2
 60 70
VL aatcgctacactggagtccctgatcgcttcactggcagtggatatgggacggatttcactttcaccatcagcactgtg
 N R Y T G V P D R F T G S G Y G T D F T F T I S T V
1F9 attcgctacactggagtccctgatcgcttcactggcagtggatatgggacggatttcactttcaccatcagcactgtg
 I R Y T G V P D R F T G S G Y G T D F T F T I S T V
 24 42 72
 80 90 100
VL caggctgaagacctggcagtttatttctgtcagcaggattatagctctcctccCACAGTGcttcagcctcctACACAAACC
 Q A E D L A V Y F C Q Q D Y S S P 7 mer 9 mer
J2 GGTTTTTGTacagccagacagtggagtactacCACTGTGgtggacgttcggtggaggcaccaagctg
 9 mer 7 mer T F G G G T K L
1F9 caggttgaagacctggcagtttatttctgtcagcagggttttagctctcctcggacgttcggtggaggcaccaagctg
 Q V E D L A V Y F C Q Q G F S S P R T F G G G T K L
 CDR3 37
 110 120 130
J2 gaaatcaaACGTAAGTagaatcc
 E I K |splice donor
CL CCTTCCTCAGGggctgatgctgcaccaactgtatccatcttcccaccatccagtgagcagttaacatctggaggtgcc
 splice accept| A D A A P T V S I F P P S S E Q L T S G G A
1F9 gaaatcaatcgggctgatgctgcaccaactgtatccatcttcccaccatccagtgagcagttaacatctggaggtgcc
 E I N R A D A A P T V S I F P P S S E Q L T S G G A
