# Supplementary material for: Structure of the Malaria Antigen AMA1 in Complex with a Growth-Inhibitory Antibody
Source: PLoS Pathog. 2007 Sep 28;3(9):e138. doi: 10.1371/journal.ppat.0030138 (PMC2323298; doi:10.1371/journal.ppat.0030138)
Supplement: Table S1 — Table of AMA1 residues that contact 1F9 showing the polymorphisms in the field, and the polymorphic difference probability (the probability that two sequences differ at a position). Table S1 also shows the 1F9-buried surface for each AMA1 residue in crystal form 2. Polymorphic residues tend to be more exposed and present a larger surface area to 1F9. (43 KB DOC) [file ppat.0030138.st001.doc]

| **Epitope**  **residue** | **Position**  **in AMA1** | Polymorphisms | **Difference**  **probability** |  | **1F9-buried area in**  **crystal form 2 (Å^2^)** | |
| --- | --- | --- | --- | --- | --- | --- |
|  |  |  |  |  |  | |
| F183 | trough | - | - |  | 22 | |
| T186 | loop Ic | - | - |  | 47 | |
| E187 | loop Ic | NEK | 0.64 |  | 18 | |
| P188 | loop Ic | - | - | 88 | |  |
| L189 | loop Ic | LPH | 0.17 | 38 | |  |
| M190 | trough | MI | 0.48 | 41 | |  |
| P192 | PAN sheet 2 | - | - | 10 | |  |
| M193 | PAN sheet 2 | - | - | 13 | |  |
| T194 | PAN sheet 2 | - | - | 7 | |  |
| D196 | loop Id | DNY | 0.40 | 48 | |  |
| E197 | loop Id | GQDHERV | 0.78 | 93 | |  |
| R199 | loop Id | - | - | 10 | |  |
| H200 | loop Id | DHLR | 0.61 | 117 | |  |
| F201 | loop Id | FLSV | 0.38 | 103 | |  |
| Y202 | trough | - | - | 25 | |  |
| K203 | loop Id | - | - | 67 | |  |
| D204 | loop Id | DN | 0.50 | 109 | |  |
| N205 | loop Id | - | - | 39 | |  |
| V208 | trough | - | - | 14 | |  |
| K209 | loop Id | - | - | 7 | |  |
| G222 | PAN helix | - | - | 10 | |  |
| N223 | PAN helix | - | - | 84 | |  |
| M224 | trough | MI | 0.03 | 25 | |  |
| I225 | loop Ie | NI | 0.39 | 77 | |  |
| N228 | loop Ie | NK | 0.10 | 47 | |  |
| K230 | loop Ie | KEQ | 0.45 | 50 | |  |
| K235 | loop Ie | - | - | 17 | |  |
